# Retrospective analysis of the impact of platinum dose reduction and chemotherapy delays on the outcomes of stage III ovarian cancer patients

**DOI:** 10.1186/s12885-015-1104-5

**Published:** 2015-03-07

**Authors:** Sigita Liutkauskiene, Rasa Janciauskiene, Kristina Jureniene, Saulius Grizas, Rasa Malonyte, Elona Juozaityte

**Affiliations:** 1 Oncology Institute of Lithuanian University of Health Sciences, Kaunas, Lithuania; 2 Institute of Biomedical Sciences of Lithuanian University of Health Sciences, Kaunas, Lithuania; 3 Clinic of Surgery of Lithuanian University of Health Sciences, Kaunas, Lithuania; 4 Medical Academy, Faculty of Medicine, Lithuanian University of Health Sciences, Kaunas, Lithuania

**Keywords:** Ovarian cancer, Chemotherapy scheme modifications, Progression free survival, Overall survival

## Abstract

**Background:**

Ovarian cancer is a common gynaecological malignancy still remaining a challenge to treat. The objective of this study was to evaluate the impact of platinum dose reduction and chemotherapy delays on progression free survival and overall survival in patients with stage III ovarian cancer and to analyze reasons for such chemotherapy scheme modifications.

**Methods:**

Medical records of patients with FIGO stage III ovarian cancer were reviewed. Inclusion criteria involved FIGO stage III epithelial ovarian carcinoma; cytoreductive surgery performed and 6 courses of platinum-based chemotherapy completed; no neoadjuvant chemotherapy applied; and no history of previous malignancies. Progression free survival and overall survival were analyzed using Kaplan-Meier and Cox proportional hazards models.

**Results:**

Significant 3.3 times higher death risk in patients who experienced only chemotherapy delays compared with patients who did not experience any chemotherapy scheme modifications was established (HR = 3.3, 95% Cl: 1.2 – 8.5, p = 0.016). Increased death risk in patients who experienced only chemotherapy delays compared with patients who experienced both chemotherapy delays and platinum dose reduction was also established (HR = 2.3, 95% Cl: 1.1 – 4.8, p = 0.021). Main reasons for chemotherapy scheme modifications (in decreasing order) were the following: neutropenia, modifications with no objective medical reasons, renal disorders, anaemia, poor performance status, gastrointestinal symptoms and neuropathy. Overall survival in patients who experienced chemotherapy scheme modifications with no objective medical reasons was non-inferior than in patients who did not experience any chemotherapy scheme modifications.

**Conclusions:**

Chemotherapy delays in patients with FIGO stage III ovarian cancer caused lower overall survival. The most common reason for chemotherapy scheme modifications was neutropenia.

## Background

Ovarian cancer is the fifth most common malignant tumor in females and the forth most common cause of female deaths from malignant tumors in the world [1]. In 2012 in Europe 65 538 new ovarian cancer cases were diagnosed and 42 704 deaths from ovarian cancer were registered. Meanwhile Lithuania ranks 5th among all European countries (incidence is 16.2 cases/100 000 females and mortality is 11.9/100 000 females) [2]. Early diagnosis of ovarian cancer is still difficult due to absence of specific clinical signs or asymptomatic course of the disease therefore advanced cancer is being diagnosed in majority of patients [3]. Association between the diameter of the postoperative residual tumor mass and survival was first discovered by C. T. Griffths in 1975 [4] and later proved by other studies [5,6] and these findings were the cornerstone of the progress in ovarian cancer treatment. Progress in treatment was also essentially influenced by the results of studies analyzing the effectiveness of various chemotherapy schemes carried out in the recent decades resulting in employing platinum - based adjuvant chemotherapy as main method of systemic treatment of advanced ovarian cancer [7]. Despite the fact that disease progression after primary chemotherapy is observed in approximately two thirds of patients [8], five-year survival rate is improving from 5–17% decades ago to 48% and more nowadays according to some researchers [9]. According to the data of the Nordic countries obtained during 40 years observation period survival rates of women with ovarian cancer improved 10–15% from 1964 to 2003 [10]. Impact of tumor stage, histology, grade and patients’ age on clinical outcomes is well established [11,12]; however few studies analyzed impact of chemotherapy delays and dose reduction on progression free survival and overall survival [13]. Therefore the purpose of this study is to establish influence of platinum dose reduction and chemotherapy delays on progression free survival and overall survival in women with stage III ovarian cancer and to analyze reasons for such chemotherapy scheme modifications.

## Methods

This study was approved by the Bioethics Center of Lithuanian University of Health Sciences. Medical records of patients with International Federation of Gynecology and Obstetrics (FIGO) stage III ovarian cancer treated in the Afilliate of Lithuanian University of Health Sciences Kaunas Oncology Hospital were analyzed.

Inclusion criteria of the study were the following: 1) patients with FIGO stage III epithelial ovarian carcinoma diagnosed in 2004–2008; 2) cytoreductive surgery performed and 6 courses of platinum-based chemotherapy completed; 3) medical records maintain comprehensive data on treatment and follow-up; 4) no history of previous malignancies. Exclusion criteria were the following: 1) neoadjuvant chemotherapy applied; 2) less than 6 courses of chemotherapy or more than 6 courses of chemotherapy completed; 3) medical records maintain incomplete data on treatment or follow-up; 4) history of previous malignancy.

Among all patients treated with platinum – based chemotherapy only those who received 6 courses were selected, because usually 6 cycles of treatment is recommended as a standard regimen; what is more this decision was taken in order to avoid possible differences in patients outcomes influenced by unequal number of chemotherapy cycles given.

**Figure 1:**
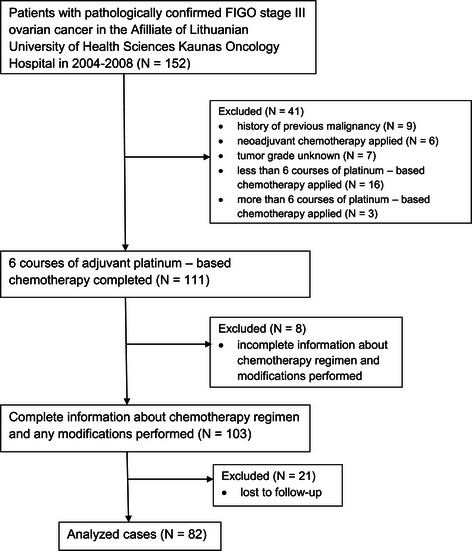
**Flow chart of cases used for analysis.** The whole process of identification of the patients that meet study criteria is shown in this chart. 82 patients were used for final analysis.

The process of cases selection is shown in Figure 1.

The following data necessary for this study were collected: patients age at the time of diagnosis, height and weight, tumor histology and grade, date of cytoreductive surgery, date of disease progression, date of last visit, date of death. Platinum response status was evaluated (platinum resistant tumor: disease progression occurred within 6 months after completion of primary chemotherapy; platinum sensitive tumor: disease progression occurred after more than 6 months after completion of primary chemotherapy). Platinum dose reduction was defined as overall dose reduction more than 5 percent; chemotherapy delays were defined as number of delayed days exceeding 10 days. 5 percent as the cutoff for dose reduction and 10 days as the cutoff for the delay were defined because such were minimal modifications observed in our study. Reason for every single platinum dose reduction and chemotherapy delay was established. It should be noted that no granulocyte colony stimulating factors were administered to patients under investigation.

### Statistical analysis

The major goals of the study were to assess the impact of platinum dose reduction and chemotherapy delays on survival outcomes – progression free survival (PFS) and overall survival (OS). PFS was defined as a period between the date of cytoreductive surgery and date when tumor progression was defined or date of the last known follow up for patients without disease progression. OS was defined as the duration between the date of cytoreductive surgery and death, with those alive censored at the last known follow up.

The Kaplan-Meier method was used for single variable survival data analysis. Differences of the survival rate were determined using the log-rank test.

Cox’s proportional hazards regression model was used to conduct multivariate analysis. The proportional hazards assumption was examined by log (−log) plot of survival. The results are presented as medians of survival, HR and 95% confidence intervals.

Comparison among subgroups was performed using ANOVA for continuous covariates and Fisher’s exact test for categorical variables.

Level of significance for statistical tests was 0.05. All p - values presented are two – sided. The SPSS software version 22 was used for statistical analysis.

## Results

Eighty-two eligible patients were analyzed. For all patients median progression free survival was 15.4 months (95% Cl: 9.3 - 21.4 months), and median overall survival was 32.1 months (95% Cl: 25.5 – 38.7 months).

Patients were divided into four chemotherapy delay/platinum reduction groups. There was no platinum dose reduction or chemotherapy delays in 15 patients (18.3%). In 67 patients (81.7%) chemotherapy scheme was modified: 11 patients (13.4%) experienced only platinum dose reduction, 12 patients (14.6%) experienced only chemotherapy delays and 44 patients (53.7%) experienced both platinum dose reduction and chemotherapy delays.

Age at the time of diagnosis was significantly different in these four groups (p = 0.001); other characteristics were similar (Table 1).

**Table 1:** **Patients groups characteristics**

	No platinum reduction or delays	Platinum reduction only	Delays only	Both Platinum reduction and delays
	(N = 15)	(N = 11)	(N = 12)	(N = 44)
Age, mean (range)	46.6 (22–67)	52.0 (42–65)	60.8 (42–82)	59.7 (40–82)
BMI, mean (range)	24.9 (16.1–46.1)	25.1 (18.2–33.7)	26.5 (19.5–39.5)	26.9 (16.7–38.2)
(kg/m^2^)				
Histology				
Serous	13 (86.6%)	11 (100%)	10 (83.4%)	32 (72.7%)
Mucinous	1 (6.7%)	0 (0%)	1 (8.3%)	4 (9.1%)
Endometrioid	0 (0%)	0 (0%)	1 (8.3%)	1 (2.3%)
Clear cell	0 (0%)	0 (0%)	0 (0%)	2 (4.5%)
Mixed	1 (6.7%)	0 (0%)	0 (0%)	5 (11.4)
Tumor grade				
Grade 1	0 (0%)	1 (9.1%)	1 (8.3%)	6 (13.6%)
Grade 2	11 (73.3%)	8 (72.7%)	9 (75.0%)	24 (54.6%)
Grade 3	4 (26.7%)	2 (18.2%)	2 (16.7%)	14 (31.8%)

(N = 82).

The impact of patients' age, BMI, tumor grade, histology and platinum response status to overall survival was tested conducting univariate Cox analysis. All these variables except BMI were proved to have a possible prognostic significance (p < 0.35) in univariate analysis and were adopted as covariates in multivariate Cox proportional risk model. Results of this analysis for overall survival are presented in Table 2.

**Table 2:** **Univariate and multivariate analysis of predictors for overall survival**

	Univariate model				Multivariate model			
Variables	Exp (B)	95.0% CI for Exp (B)		p	Exp (B)	95.0% CI for Exp (B)		p
		Lower	Upper			Lower	Upper	
Platinum response status (resistant versus sensitive)	3.412	2.002	5.817	<0.001	3.169	1.787	5.620	<0.001
Chemoterapy scheme modification				0.035				0.050
No Platinum reduction or delay	1				1			
Both Platinum reduction and delay	1.380	0.658	2.896	0.394	1,404	0.615	3.205	0.420
Platinum reduction only	1.388	0.550	3.500	0.487	2,054	0.755	5.586	0.159
Delay only	3.359	1.375	8.204	0.008	3.262	1.248	8,527	0.016
Tumor grade	1.386	0.908	2.115	0.130	1.547	0.936	2.556	0.089
Patients’ age				0.225				0.260
<=50	1				1			
51–65	0.979	0.531	1.805	0.945	0.723	0.362	1.445	0.359
>65	1.591	0.865	2.926	0.135	1.327	0.653	2.695	0.434
histology (serous versus other)	1.393	0.706	2.750	0.339	1.076	0.518	2.234	0.844
BMI	1.012	0.973	1.053	0.539				
					HR			p
Delay only versus both reduction and delay					2.323	1.135	4.753	0.021

This Cox proportional hazards model for overall survival with adjustment for patients’ age, tumor grade, histology and platinum response status revealed statistically significant overall survival difference in patients who experienced only chemotherapy delays compared with patients who did not experience neither chemotherapy delays nor platinum dose reduction, e. g., death risk was 3.3 times higher in patients who experienced only chemotherapy delays compared with patients who did not experience any modifications (HR = 3.3, 95% Cl: 1.2 – 8.5, p = 0.016). Significantly increased death risk in patients who experienced only chemotherapy delays compared with patients who experienced both chemotherapy delays and platinum dose reduction was also established (HR = 2.3, 95% Cl: 1.1 – 4.8, p = 0.021).

**Figure 2:**
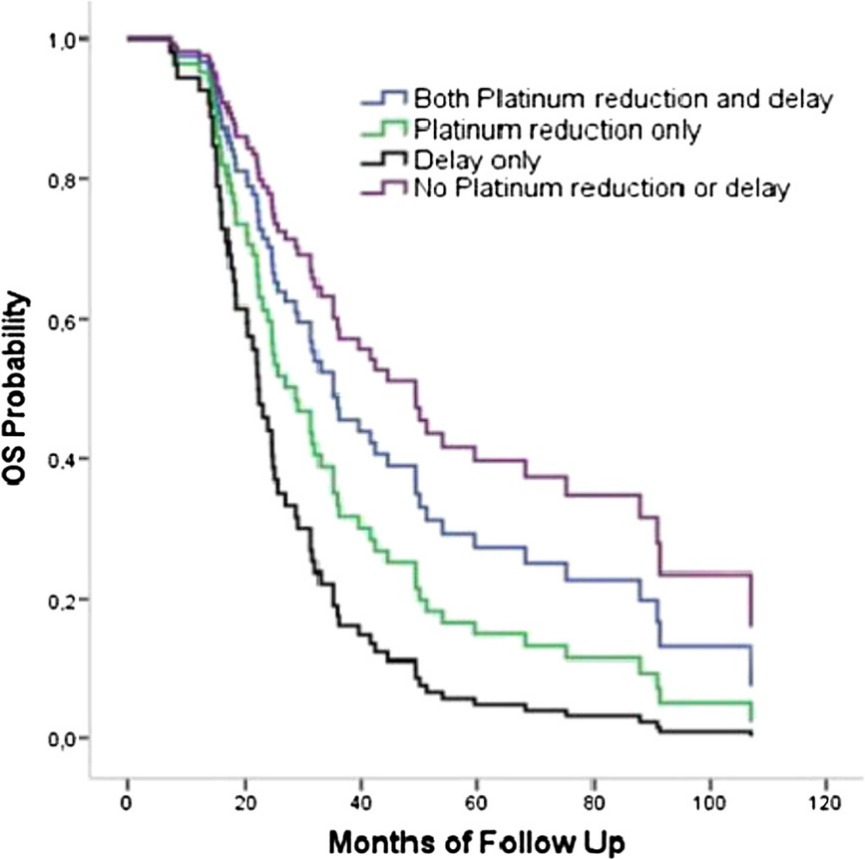
**Overall survival: adjusted for age, tumor grade, histology and platinum response status.** Cox proportional hazards model when impact of tumor histology, grade, patients’ age and platinum response status were adjusted showed that death risk was 3.3 times higher in patients who experienced only chemotherapy delays compared with patients who experienced no chemotherapy delays or platinum dose reduction (HR = 3.3, 95% Cl: 1.2 – 8.5, p = 0.016) and also showed that death risk was 2.3 times higher in patients who experienced only chemotherapy delays compared with patients who experienced both chemotherapy delays and platinum dose reduction (HR = 2.3, 95% Cl: 1.1 – 4.8, p = 0.021).

Survival curves for overall survival adjusted for patients’ age, tumor grade, histology and platinum response status is shown in Figure 2.

According to Cox proportional hazards model for progression free survival constructed identically to above-mentioned model, there were no statistically significant differences of progression free survival between the same four patients groups, the overall p = 0.35.

Median progression free survival and median overall survival in these groups are presented in Table 3.

**Table 3:** **Unajusted median progression free and overall survival (95% Cl)**

	N	PFS (months)	OS (months)
**No platinum reduction or delays**	15	15.4 (9.3 – 21.4)	35.8 (16.1 – 55.6)
**Platinum reduction only**	11	13.1 (10.7 – 34.5)	28.8 (20.5 – 37.2)
**Delays only**	12	10.8 (4.2 – 17.4)	15.8 (11.9–19.8)
**Both Platinum reduction and delays**	44	18.3 (10.9 – 25.7)	39.6 (26.2 – 52.4)

**Figure 3:**
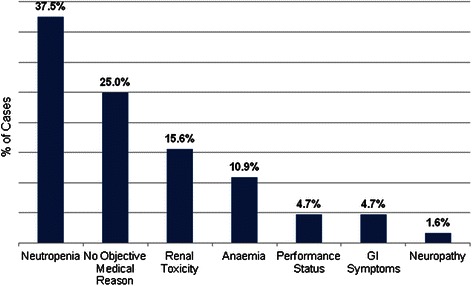
**Reasons for chemotherapy scheme modifications.** Reason for every single platinum dose reduction and chemotherapy delay was established. The most common of them from most to least frequent were neutropenia, modifications with no objective medical reasons, renal disorders, anaemia, poor performance status, gastrointestinal (GI) symptoms and neuropathy.

This study showed that main reasons for chemotherapy scheme modifications were the following: neutropenia (37.5%), renal disorders (15.6%), anaemia (10.9%), poor performance status (4.7%), gastrointestinal symptoms (4.7%), and neuropathy (1.6%). Modifications with no objective medical reason accounted for 25.0%. (Figure 3). When medical records contained no medical reason that could predetermine platinum dose reduction or chemotherapy delay, such modification was considered to have no objective medical reason. Considering this reason was the second most frequent among all reasons of chemotherapy scheme modifications, overall survival curve of patients who experienced modifications without objective medical reasons was compared with overall survival curve of patients who had no modifications. Using Kaplan-Meier method it was not proved that chemotherapy scheme modifications without objective medical reason caused lower overall survival compared with patients who had no chemotherapy scheme modifications, long-rank test p = 0.815. When chemotherapy scheme was modified with no objective medical reason, median progression free survival was 26.4 months (95% Cl: 16.7 - 36.2 months) and median overall survival was 51.2 months (95% Cl: 23.4 - 78.9 months).

## Discussion

The main objective of this study was to evaluate impact of the optimum first-line chemotherapy on clinical outcomes of patients with FIGO stage III ovarian cancer. Although many previous studies analyzing effect of chemotherapy schemes intensity on survival did not establish any advantages of more intense treatment compared with less intense treatment [14-16], an impact of modifications of standard chemotherapy schemes, e.g., dose reduction and chemotherapy delays, on survival outcomes is still unclear because results of similarly designed studies are contradictory. To characterize modifications of chemotherapy schemes, the term relative dose intensity (RDI) is also used in scientific literature; RDI is calculated as delivered dose intensity (total delivered dose divided by actual time required to complete therapy) divided by standard dose intensity (standard dose divided by planned time to complete therapy). The impact of both factors forming the concept of RDI, i.e., dose reduction and chemotherapy delays, on survival were analyzed in our study. C. I. Nagel et al. did not establish statistically significant survival difference between patients who underwent modifications of chemotherapy scheme and those that did not [13]. However J. M. Fauci et al. in their study on the effect of RDI for the efficacy of treatment of all stages of ovarian cancer make a conclusion that dose reduction and chemotherapy delays are important prognostic factors for survival and these modifications should be minimized [17]. R. K. Hanna et al. present similar results: reduced RDI exerted negative effect on overall survival of patients with stage III-IV ovarian cancer. No impact of chemotherapy scheme modifications on progression free survival was found in the aforementioned study by R. K. Hanna et al. [18], and our study demonstrates the same result. It was established in our study that death risk was statistically significant 3.3 times higher (p = 0.016) in patients who experienced only chemotherapy delays compared with patients who did not experience any chemotherapy scheme modifications. These results essentially confirm conclusions of aforementioned studies carried out by J. M. Fauci et al. and R. K. Hanna et al. concerning negative impact of chemotherapy scheme modifications on patients overall survival. However, our study also established 2.3 times higher death risk (p = 0.021) in patients who experienced only chemotherapy delays compared with patients who experienced both chemotherapy delays and platinum dose reduction. This result was unexpected for us because it means that extension of the intervals between chemotherapy courses (extends also overall duration of chemotherapy) without dose reduction may predetermine shorter overall survival in patients with FIGO stage III ovarian cancer. Such trend could be caused by the toxicity of chemotherapy related to patient’s individual sensitivity to chemotherapy. This result confirms the general principle of optimum chemotherapy: when grade III-IV adverse reaction occurs, chemotherapeutic drugs dose must be reduced by 20–25 percent due to threatening complications or adverse reactions to chemotherapy should be managed and treated appropriately. It should be emphasized that main reason for chemotherapy scheme modifications in our study was neutropenia, and colony-stimulating factors were not administered to patients of our study. On the other hand, similar study on RDI significance for clinical outcomes in patients with metastatic breast cancer carried out by S. Loibl et al. also showed an unexpected result: optimal RDI was related to shorter progression free survival. This result was associated with early progression of the disease when disease progression or patient death occurred before 3rd course of chemotherapy. When data of early disease progression were excluded from the analysis, positive optimum RDI impact on overall survival was established [19]. However, publication authors do not indicate how many patients died before 3rd course of chemotherapy and what were the causes of deaths; these deaths could be possibly caused by chemotherapy toxic effects. It is clear today that clinical study participants are usually patients with better performance status without concomitant diseases or with clinically insignificant concomitant diseases, and therefore optimum chemotherapy treatment is administered to these patients. However, in everyday clinical practice chemotherapy dose reduction and chemotherapy delays are frequent, and previous surgery, doctor’s experience, adequate assessment of patients’ performance status, other treatment lines and many social factors affects clinical outcomes. It is also worth to state that in our study in all patients who experienced chemotherapy delays total number of delayed days exceeded 10 days and this factor could contribute lower survival as in C. I. Nigel et al. study that did not established negative impact of delays on survival, total number of delayed days was lower than ten days in a significant part of patients [13].

Neutropenia was the main reason for chemotherapy delays and platinum dose reduction (37.5%). Neutropenia-related complications often demands not only specific treatments but also chemotherapy scheme modifications: prospective cohort study published in 2014 showed that 60% of patients with stage IV solid tumors needed RDI reduction. Patients with stage IV ovarian cancer composed 8% of all participants of this study [20]. Eisenhauer et al. presented the following reasons for chemotherapy scheme modifications in patients with stage IIIC-IV ovarian cancer: myelosuppression was the main reason in patients under 65 years, and poor performance status was the main reason in patients over 65 years. Chemotherapy scheme modification because of poor performance status was observed in 5.6% of all modifications made in our study [21]. Our data showed that 25.0% of chemotherapy scheme modifications was not clinically grounded. This was specified as modification with no objective medical reason, and this notion involves various social circumstances, transportation issues interfering patient’s admission at scheduled date, and unmotivated doctor’s decision to reduce recommended dose. We failed to prove statistically significant negative impact of these factors on overall survival of patients in our study.

Small subject population is limitation of this retrospective study. Possible variations of further treatment were not taken into account and this could affect results of patients survival. This study also had advantages: all patients were treated in the same healthcare institution, same methods of examination, initial treatment and follow-up were applied and discrepancies in medical documentation were avoided. Considering that only few similar studies were carried out, findings of our study are useful in clinical practice when chemotherapy scheme individualization is necessary. Results of our study will also be important in future: these are historical data worth to review when new systemic treatment or surgery innovations emerge. However, larger prospective studies are needed in order to obtain more accurate and reliable results.

## Conclusions

Results of our study showed that chemotherapy delays when treating patients diagnosed with FIGO stage III ovarian cancer were associated with lower overall survival. According to our study, the most common reason for platinum dose reduction or chemotherapy delays was neutropenia.
